# Clinical and laboratory data, radiological structured report findings and quantitative evaluation of lung involvement on baseline chest CT in COVID-19 patients to predict prognosis

**DOI:** 10.1007/s11547-020-01293-w

**Published:** 2020-10-12

**Authors:** Cappabianca Salvatore, Fusco Roberta, de Lisio Angela, Paura Cesare, Clemente Alfredo, Gagliardi Giuliano, Lombardi Giulio, Giacobbe Giuliana, Russo Gaetano Maria, Belfiore Maria Paola, Urraro Fabrizio, Grassi Roberta, Feragalli Beatrice, Miele Vittorio

**Affiliations:** 1 Division of Radiodiagnostic, “Università degli Studi della Campania Luigi Vanvitelli”, Naples, Italy; 2 Radiology Division, “Istituto Nazionale Tumori IRCCS Fondazione Pascale – IRCCS di Napoli”, Naples, Italy; 3 Diagnostic Imaging Unit, “Azienda Ospedaliera di Rilievo Nazionale Giuseppe Moscati”, Avellino, Italy; 4 Department of Medical, Oral and Biotechnological Sciences - Radiology Unit “G. D’Annunzio”, University of Chieti-Pescara, Chieti, Italy; 5 Division of Radiodiagnostic, “Azienda Ospedaliero-Universitaria Careggi”, Firenze, Italy

**Keywords:** COVID-19, Chest CT, Outcome, Regression model

## Abstract

**Objective:**

To evaluate by means of regression models the relationships between baseline clinical and laboratory data and lung involvement on baseline chest CT and to quantify the thoracic disease using an artificial intelligence tool and a visual scoring system to predict prognosis in patients with COVID-19 pneumonia.

**Materials and methods:**

This study included 103 (41 women and 62 men; 68.8 years of mean age—range, 29–93 years) with suspicious COVID-19 viral infection evaluated by reverse transcription real-time fluorescence polymerase chain reaction (RT-PCR) test. All patients underwent CT examinations at the time of admission in addition to clinical and laboratory findings recording. All chest CT examinations were reviewed using a structured report. Moreover, using an artificial intelligence tool we performed an automatic segmentation on CT images based on Hounsfield unit to calculate residual healthy lung parenchyma, ground-glass opacities (GGO), consolidations and emphysema volumes for both right and left lungs. Two expert radiologists, in consensus, attributed at the CT pulmonary disease involvement a severity score using a scale of 5 levels; the score was attributed for GGO and consolidation for each lung, and then, an overall radiological severity visual score was obtained summing the single score. Univariate and multivariate regression analysis was performed.

**Results:**

Symptoms and comorbidities did not show differences statistically significant in terms of patient outcome. Instead, SpO2 was significantly lower in patients hospitalized in critical conditions or died while age, HS CRP, leukocyte count, neutrophils, LDH, d-dimer, troponin, creatinine and azotemia, ALT, AST and bilirubin values were significantly higher. GGO and consolidations were the main CT patterns (a variable combination of GGO and consolidations was found in 87.8% of patients). CT COVID-19 disease was prevalently bilateral (77.6%) with peripheral distribution (74.5%) and multiple lobes localizations (52.0%). Consolidation, emphysema and residual healthy lung parenchyma volumes showed statistically significant differences in the three groups of patients based on outcome (patients discharged at home, patients hospitalized in stable conditions and patient hospitalized in critical conditions or died) while GGO volume did not affect the patient's outcome. Moreover, the overall radiological severity visual score (cutoff ≥ 8) was a predictor of patient outcome. The highest value of *R*-squared (*R*^2^ = 0.93) was obtained by the model that combines clinical/laboratory findings at CT volumes. The highest accuracy was obtained by clinical/laboratory and CT findings model with a sensitivity, specificity and accuracy, respectively, of 88%, 78% and 81% to predict discharged/stable patients versus critical/died patients.

**Conclusion:**

In conclusion, both CT visual score and computerized software-based quantification of the consolidation, emphysema and residual healthy lung parenchyma on chest CT images were independent predictors of outcome in patients with COVID-19 pneumonia.

## Introduction

The severe acute respiratory syndrome coronavirus 2 (SARS-CoV-2) has been indicated the virus responsible of SARS-CoV-2 disease (COVID-19), which has spread worldwide since December 2019 [1–4].

COVID-19 diagnosis is made using “reverse transcription real-time fluorescence polymerase chain reaction” (RT-PCR) test obtained by respiratory tract or blood specimens.

Triage of the COVID-19 patients is based on clinical and laboratory parameters, whilst chest imaging might be required for second-level triage by means of chest radiography as the first step and supplementary computed tomography (CT) in more severe cases or in case of discrepancy between clinical and radiographic findings [5, 6]. Although CT examinations can be used for lung involvement monitoring and several publications attempted to show that CT could identify COVID-19 viral pneumonia, the field is highly debated and several radiological organizations not have recommended the CT as a routine screening tool in the COVID-19 pneumonia [7–16]. However, CT examination was used to evaluate the grade and the extension of the viral pneumonia by COVID-19 [12, 13].

Li et al. have reported that visual quantitative analysis of CT abnormalities reproduces clinical characteristics of COVID-19 infection [10]. Moreover, lung involvement of COVID-19 pneumonia could be quantified automatically by artificial intelligence (AI) and deep learning-based algorithms on baseline chest CT [11–13]. CT quantification of healthy residual lung parenchyma was shown to be helpful either to estimate the alveolar recruitment during ventilation or to predict the patient’s prognosis of patients with acute respiratory distress syndrome [14, 15].

The aim of the study was to evaluate by means of regression models the relationships between baseline clinical and laboratory data and lung involvement on baseline chest CT, to quantify the thoracic disease using an artificial intelligence tool and to assess a visual scoring system to predict prognosis in patients with COVID-19 pneumonia.

## Materials and methods

### Patient characteristics

In relation to the ongoing epidemic emergency, the institutional review board (IRB) of “AORN Giuseppe Moscati” approved the study and waived written informed consent for this retrospective study that evaluated anonymized data and involved no potential risk to patients. Our cohort was composed of 103 (41 women and 62 men; 68.8 years of mean age—range, 29–93 years) subjected to the RT-PCR test for suspicious COVID-19 disease, between March 6, 2020, and March 31, 2020. The virus investigation for etiological diagnosis was executed by the current gold standard test in the clinical laboratory of “AORN Giuseppe Moscati.”

Therefore, chest CT was performed based on high clinical suspicion in addition to clinical and laboratory findings recording in a setting of high pre-test probability of COVID-19.

**Fig. 1:**
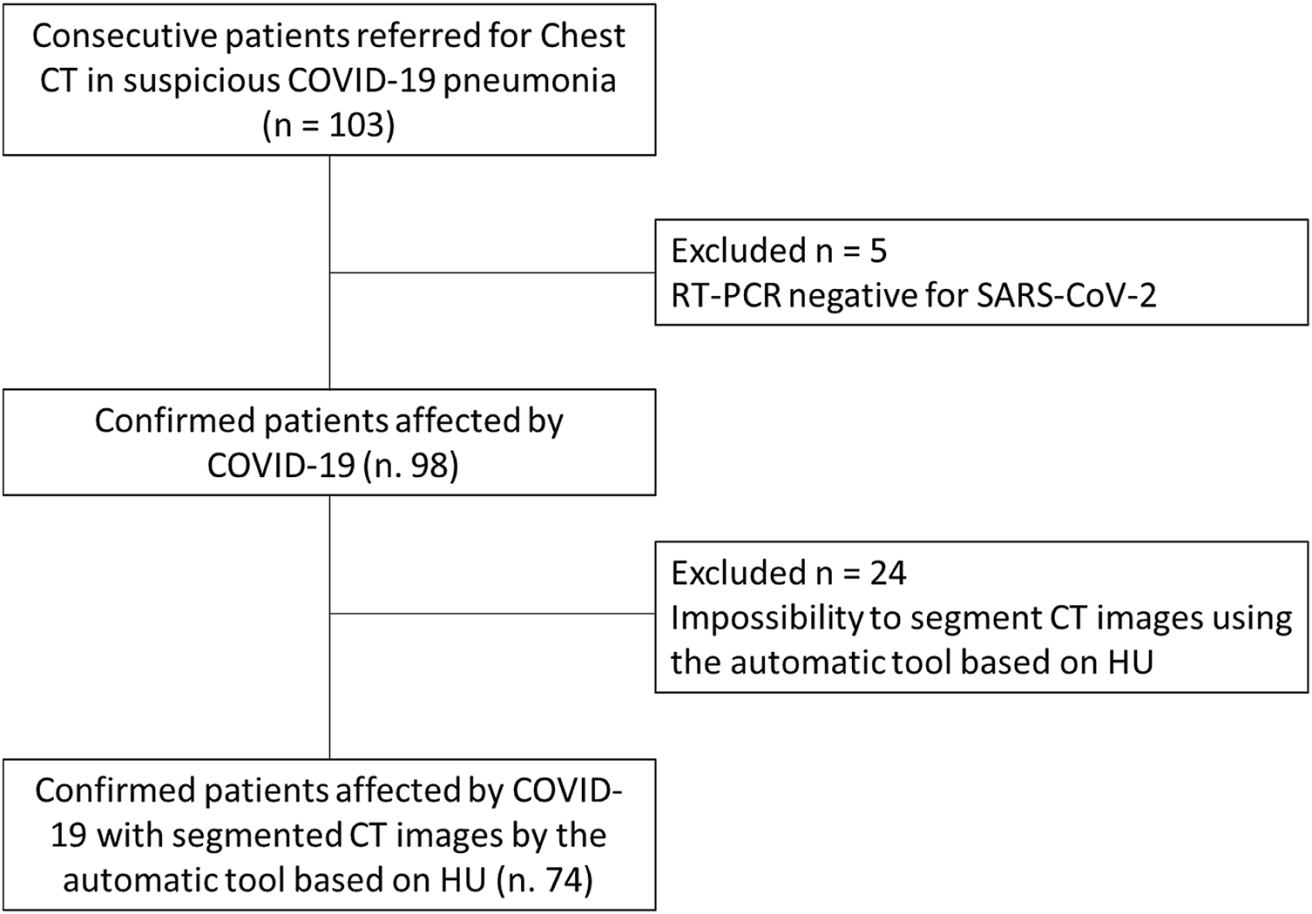
Diagram showing the patient selection process. CT, computed tomography; COVID-19, severe acute respiratory syndrome coronavirus 2 (SARS-CoV-2) disease; RT-PCR, reverse transcription polymerase chain reaction; HU: Hounsfield unit

In order to select chest CT scans for analysis, our exclusion criteria were RT-PCR for SARS-CoV-2 that was ultimately determined to be negative. Figure 1 shows the patient's enrollment flowchart.

Clinical and laboratory findings of each patient were recorded at admission. All CT examinations were performed within 2 days from the clinical evaluation and laboratory findings.

Patients were categorized in 3 groups: patients discharged at home, patients hospitalized in stable conditions and patient hospitalized in critical conditions or who died.

### CT technique

CT scan was performed at the time of patient admission in hospital. All scans were performed with the patient in the supine position during inspiration without intravenous contrast on two CT scanners (SOMATOM Sensation 16, Erlangen, Germany, and Toshiba Aquilion 64 Slices, Tokyo, Japan) dedicated to COVID-19 patients. The scanning range was from the apex to lung base. All images were obtained with a standard dose scanning protocol, reconstructed at 1.0 mm slice thickness, with 1 mm increment, 512 mm × 512 mm, 120 kV and 100–250 mAs. Images were reconstructed with a sharp reconstruction kernel for parenchyma (B80f on Somatom Sensation and FC13 on Toshiba). Lung window setting was with a window level of − 600 Hounsfield units (HU) and window width of 1600 HU.

### CT evaluation

All chest CT examinations were reviewed using a structured report defined by Italian Society of Medical Radiology and Interventional Radiology (SIRM, Milan, Italy) in collaboration with the Exprivia Healtcare company (Bari, Italy) [17]. The presence of GGO and consolidations were assessed by defining their localization (unilateral and bilateral), axial distribution (diffuse, predominantly peripheral, mainly central, declivous, anti-declivous), distribution on the cranio-caudal plane (diffuse, multifocal/patching, prevalent in the upper lobes, prevalent in the lower lobes, gravitational) and the site. The presence or absence of alterations such as septal thickening, crazy paving, “reversed halo” sign and the presence or absence of nodules and micronodules, pleural effusion, pericardial effusion and mediastinal lymphadenopathies was also assessed.

Finally, two expert radiologists in consensus, blinded to clinical/laboratory and RT-PCR findings, attributed at the CT pulmonary involvement by GGO and consolidations a radiological severity visual score using a scale of 5 levels: none (0%), mild (1–25%), moderate (26–50%), severe (51–75%) and critic (76–100%) involvement for each lung. Then, the radiological severity scores separately attributed for GGO and consolidation for each lung were summed obtaining an overall radiological severity visual score on a scale ranging from 0 to 16: none (0), mild (1–4), moderate (5–8), severe (9–12) and critic (13–16).

### CT post-processing

**Fig. 2:**
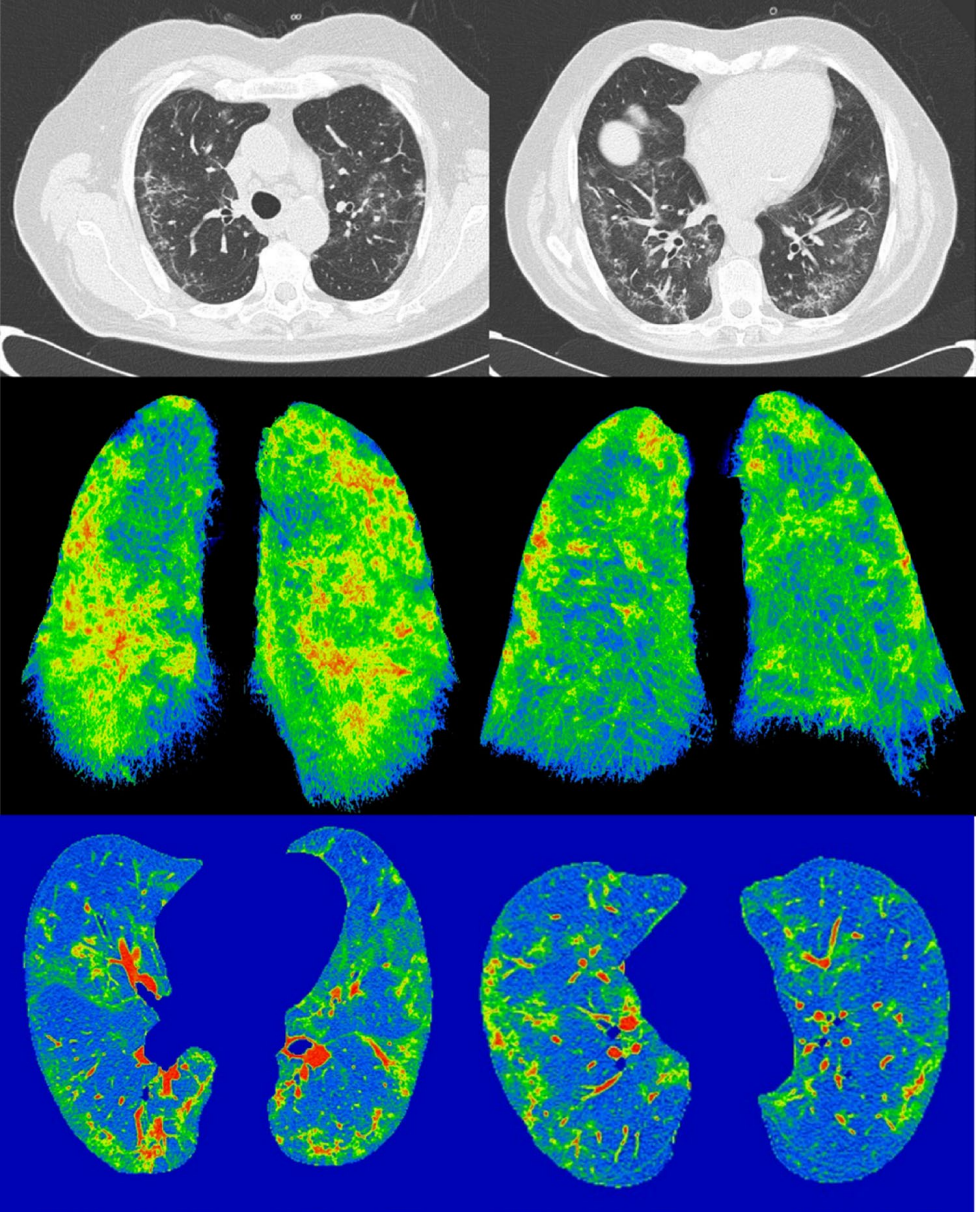
Example of lung volumes calculated by automatic segmentation tool on CT images by pre-setting a threshold value of Hounsfield Units and a color in order to obtain a segmentation of both lungs and a quantitative evaluation of emphysema (− 1024/− 977; blue), healthy residual lung parenchyma (− 977/− 703; yellow), GGO (− 703/− 368; pink) and consolidation (− 100/5; red)

DICOM data were transferred into a PACS workstation (AW SERVER 3.2 ext 3.0, of GE Healthcare, Chicago, Illinois, USA), and CT images were evaluated using a clinically available Artificial Intelligence tool (Thoracic VCAR of GE Healthcare) to obtain an automatic segmentation and quantification of the thoracic disease. The software is a CE marked medical device authorized to document emphysema. The software provides automatic segmentation of the lungs and automatic segmentation and tracking of the airway tree. It provides the classification of voxels based on Hounsfield Units and a color-coded display of the thresholds within a segmented region. This software is designed to quantify pulmonary emphysema in patients with chronic obstructive pulmonary disease. In our case, we analyzed the CT scans of patients with suspicious COVID-19 pneumonia by pre-setting threshold values of Hounsfield Unit in order to obtain a segmentation of both lungs and a quantitative evaluation of Emphysema (− 1024/− 977; blue) [18], residual healthy lung parenchyma (− 977/− 703; yellow) [19], GGO (− 703/− 368; pink) and consolidation (− 100/5; red) [20–22]. Finally, volumes for both right and left lungs were calculated (Fig. 2).

### Clinical and laboratory findings recording

Clinical and laboratory findings recording included symptoms, comorbidities and laboratory results.

Among symptoms were reported fever, cough, respiratory failure, dyspnea, asthenia and other (arthralgia, diarrhea, leukopenia, nausea).

Among comorbidities were reported hypertension, diabetes, neurological, cardiovascular, oncological and pulmonary.

Among laboratory findings were obtained SpO2 (%), HS CRP (mg/dL), leukocyte (10^3^ µL), lymphocytes (%), neutrophils (%), procalcitonin (ng mL) cutoff 0.08 (%), LDH (IU/L), d-dimer (mg/L), lactates (IU/L), troponin (mg/mL), creatinine (mg/dL), azotemia (mg/dl), glicemia (mg/dl), ALT (IU/L), AST (IU/L), bilirubin (mg/dl), prothrombin time (s), partial thromboplastin time (s), fibrinogen (mg/dl), sodium (mmol/L).

### Statistical analysis

Continuous data were expressed in terms of median value and range. Categorical data are expressed as counts and percentages. Kruskal–Wallis test was used to verify differences between groups of continuous variables. Chi-square test with Yates’s correction was used to assess statistically difference between percentage values among groups. Spearman correlation coefficient was calculated to assess the correlation between the volumes of residual healthy lung parenchyma, of GGO and consolidations with the radiological severity visual score.

The outcome was defined by discharging at home, hospitalization in stable conditions and hospitalization in critical conditions or death. Categories from continuous variables were obtained using as threshold the median value of the overall sample. Univariable and backward stepwise multivariable logistic regression analysis was used to test the association between potential predictors and the outcome. Factors for which *p* values were less than 0.1 in univariable analysis were used as candidate variables for multivariable approach.

Therefore, was obtained a model using only clinical parameters, a model using CT parameters and a model using clinical parameters combined to CT volumes. The *R*-squared (*R*^2^) of each model was then reported for comparison.

Receiver operating characteristic (ROC) curve analysis was performed for each model, and the area under the ROC (AUC) was used to assess the performance of the discrimination models based on independent predictors.

*p* value < 0.05 was considered significant for all tests.

All analyses were performed using Statistics Toolbox of MATLAB R2007a (The Math-Works Inc., Natick, MA, USA).

## Results

### Patient demographics, clinical and laboratory findings

Demographics, clinical and laboratory findings are reported in Table 1. The analysis included 98 patients (median age, 61 years old; range 23–91 years old), 39/98 (39.80%) were females. Five patients were excluded for negative result at RT-PCR test (Fig. 1). No symptoms (fever, cough, respiratory failure, dyspnea or other including arthralgia, diarrhea, leukopenia, nausea), no comorbidities (hypertension, diabetes, neurological, cardiovascular, oncological, pulmonary) determined differences statistically significant in terms of patient outcome (Table 1). Among admission laboratory findings, SpO2, high sensitivity C-reactive protein (HS CRP), leukocyte count, neutrophils percentage value, lactate dehydrogenase value (LDH), d-dimer, troponin, creatinine and azotemia, ALT, AST and bilirubin values showed differences statistically significant compared to patient outcome (Table 1). SpO2 was significantly lower in patients hospitalized in critical conditions or died while age, HS CRP, leukocyte count, neutrophils percentage value, LDH, d-dimer, troponin, creatinine and azotemia, ALT, AST and bilirubin values were significantly higher (Table 1).

**Table 1:** Patient's demographics, comorbidities, symptoms and laboratory findings at admission

	All patients (*n*, 98)	Discharged at home (*n* = 39)	Hospitalized and stable (*n* = 35)	Hospitalized in serious condition or died (*n* = 24)	*p* value	Effect
Age	61.0 (23.0–91.0)	56.0 (26.0–83.0)	62.0 (23.0–91.0)	78.50 (48.0–89.0)	< **0**.**001**	**↑**
*Gender*						
Male	59 (60.20%)	22 (56.41%)	21 (60.0%)	16 (66.7%)	> 0.05	
Female	39 (39.80%)	17 (43.59%)	14 (40.0%)	8 (33.3%)		
*Symptoms*						
Fever	90 (91.83%)	36 (92.31%)	35 (100.0%)	19 (79.17%)	> 0.05	
Cough	69 (70.40%)	30 (76.92%)	24 (68.57%)	15 (62.50%)	> 0.05	
Respiratory failure	9 (9.18%)	1 (2.56%)	3 (8.57%)	5 (20.83%)	> 0.05	
Dyspnea	52 (53.06%)	22 (56.41%)	17 (48.57%)	13 (54.17%)	> 0.05	
Asthenia	11 (11.22%)	7 (17.95%)	3 (8.57%)	1 (4.17%)	> 0.05	
Other (arthralgia, diarrhea, leukopenia, nausea)	8 (8.16%)	3 (7.69%)	4 (11.42%)	1 (4.17%)	> 0.05	
*Comorbidities*						
Hypertension	42 (42.86%)	18 (46.15%)	11 831.43%)	13 (54.17%)	> 0.05	
Diabetes	12 (12.24%)	5 (12.82%)	4 (11.42%)	3 (12.50%)	> 0.05	
Neurological	9 (9.18%)	3 (7.69%)	2 (5.71%)	4 (16.17%)	> 0.05	
Cardiovascular	5 (5.10%)	0 (0.0%)	1 (2.86%)	4 (16.17%)	> 0.05	
Oncological	9 (9.18%)	3 (7.69%)	3 (8.57%)	3 (12.50%)	> 0.05	
Pulmonary	8 (8.16%)	2 (5.13%)	2 (5.71%)	4 (16.17%)	> 0.05	
*Laboratory findings*						
SpO2 (%)	93 (72–99)	94 (81–99)	94 (80–98)	89 (72–95)	< **0**.**001**	**↓**
HS CRP (mg/dL)	8.41 (0.04–32.90)	7.38 (0.04–32.0)	7.4 (0.25–24.35)	14.65 (2.37–27.0)	< **0**.**01**	**↑**
Leukocyte (10^3^ µL)	6.20 (1.60–25.10)	6.40 (2.70–21.0)	5.70 (2.60–9.20)	6.45 (1.60–25.10)	**0**.**03**	**↑**
Lymphocytes (%)	15.2 (2.40–51.40)	17.20 (4.10–44.60)	14.60 (6.60–51.40)	11.20 (2.40–24.0)	> 0.05	
Neutrophils (%)	77.55 (35.20–95.40)	74.90 (44.70–91.50)	78.70 (35.20–87.90)	81.85 (60.20–95.40)	**0**.**05**	**↑**
Procalcitonin (ng mL) cutoff 0.08 (%)	0.08 (0.01–15.80)	0.08 (0.01–1.29)	0.08 (0.02–15.80)	0.30 (0.03–6.60)	> 0.05	
LDH (IU/L)	323.0 (112.0–156.6.0)	309.0 (112.0–687.0)	309.0 (142.0–541.0)	393.0 (194.0–1566.0)	< **0**.**001**	**↑**
d-dimer (mg/L)	0.96 (0.21–35.0)	0.87 (0.24–8.0)	0.60 (0.26–2.94)	1.43 (0.21–35.0)	**0**.**01**	**↑**
Lactates (IU/L)	1.20 (0.0–11.0)	1.10 (1.0–2.0)	1.10 (0.0–2.0)	1.50 (1.0–11.0)	> 0.05	
Troponin (mg/mL)	9.90 (0.0—297.0)	7.30 (0.0–59.0)	6.05 (1.0–84.0)	23.75 (1.0–297.0)	< **0**.**001**	**↑**
Creatinine (mg/dL)	0.90 (0.50–6.40)	0.90 (0.50–1.80)	0.77 (0.53–2.40)	1.19 (0.70–6.40)	** < 0**.**001**	**↑**
Azotemia (mg/dL)	35.0 (16.0–409.0)	32.50 (17.0–126.0)	30.0 (16.0–71.0)	57.0 (25.0–409.0)	** < 0**.**001**	**↑**
Glicemia (mg/dL)	106.0 (68.0–705.0)	105.0 (68.0–705.0)	103.0 (74.0–233.0)	118.0 (87.0–342.0)	> 0.05	
ALT (IU/L)	29.0 (7.0–379.0)	21.5 (10.0–152.0)	29.0 (9.0–120.0)	32.50 (7.0–379.0)	**0**.**04**	**↑**
AST (IU/L)	38.0 (12.0–407.0)	32.50 (12.0–116.0)	33.0 (14.0–98.0)	51.0 (14.0–407.0)	< **0**.**01**	**↑**
Bilirubin (mg/dL)	0.50 (0.10–4.30)	0.50 (0.10–1.20)	0.50 (0.20–1.20)	0.55 (0.20–4.30)	**0**.**05**	**↑**
Prothrombin time (s)	8.99 (7.40–28.78)	8.89 (8.16–11.25)	9.24 (7.40–26.14)	9.13 (8.34–28.78)	> 0.05	
Partial thromboplastin time (s)	32.20 (20.0–89.50)	31.25 (20.0–38.50)	32.40 (25.40–49.40)	33.35 (23.80–89.50)	> 0.05	
Fibrinogen (mg/dL)	510.0 (99.0–905.0)	487.0 (240.0–905.0)	525.0 (257.0–830.0)	508.5 (99.0–835.0)	> 0.05	
Sodium (mmol/L)	137.0 (123.0–152.0)	136.0 (123.0–142.0)	138.0 (131.0–152.0)	136.50 (125.0–152.0)	> 0.05	

Categorical variables are expressed as counts and percentage in parenthesis. Continuous variables are shown as median and range in parenthesis

Significant *p* values (< 0.05) are showed in bold type

*HS-CRP* High sensitivity C-reactive protein, *LDH* lactate dehydrogenase

### CT findings

CT findings are summarized in Table 2. GGO with or without consolidations was the main CT pattern. GGO alone was present in 10/98 (10.2%) patients, consolidations alone were present in 2/98 (2.0%) patients while a combination of GGO and consolidations was found in 86/98 (87.8%) patients. CT COVID-19 disease was prevalently bilateral in 76/98 (77.6%) of patients with peripheral distribution in 73/98 (74.5%) of patients and multiple lobes localizations in 51/98 (52.0%) cases. Moreover, crazy paving pattern was found in 55/98 (56.1%) patients and “reversed halo” sign in 20/98 (20.4%) patients (Table 2). These CT findings did not show differences statistically significant at univariable analysis among patients groups depending on the outcome because similar prevalence was reported in the three groups (patients discharged at home, patients hospitalized in stable conditions and patient hospitalized in critical conditions or who died). Moreover, also at multivariate analysis were not obtained significant results depending on the outcome. However, the overall radiological severity visual score (cutoff ≥ 8) was a predictor of patient outcome.

**Table 2:** Computed tomography findings

	All patients (*n*, 98)	Discharged at home (*n* = 39)	Hospitalized and stable (*n* = 35)	Hospitalized in serious condition or died (*n* = 24)	*p* value	Effect
Presence of GGO ± consolidation	86 (87.76%)	35 (89.74%)	32(91.43%)	19 (79.17%)	> 0.05	
Bilateral extension	76 (77.55%)	31 (79.49%)	28 (80.0%)	17 (70.83%)	> 0.05	
Peripheral distribution	73 (74.49%)	31 (79.49%)	28 (80.0%)	14 (58.33%)	> 0.05	
multifocal/patching	48 (48.98%)	23 (58.97%)	17 (48.57%)	8 (33.33%)	> 0.05	
Multiple lobes localization	51 (52.04%)	22 (56.41%)	17 (48.57%)	12 (50.0%)	> 0.05	
Crazy paving pattern	55 (56.12%)	23 (58.97%)	21 (60.0%)	11 (45.84%)	> 0.05	
“Reversed halo” sign	20 (20.41%)	11 (28.21%)	8 (22.86%)	1 (4.17%)	> 0.05	

	All patients (*n*, 64)	Discharged at home (*n* = 28)	Hospitalized and stable (*n* = 21)	Hospitalized in serious condition or died (*n* = 15)	*p* value	Effect
*Volumes*						
Residual healthy lung parenchyma (%)	70.20 (27.0–94.20)	72.80 (41.0–92.90)	73.20 (28.20–94.20)	55.90 (27.0–86.50)	**0**.**02**	**↓**
GGO (%)	22.80 (4.10–55.50)	23.0 (6.30–34.10)	19.80 (4.10–47.20)	26.90 (8.70–55.50)	> 0.05	
Consolidation (%)	1.40 (0.30–13.20)	1.30 (0.30–7.10)	1.2 (0.40–5.40)	3.70 (0.80–13.20)	**0**.**02**	**↑**
Emphysema (%)	0.10 (0.0–11.10)	0.0 (0.0–0.90)	0.30 (0.0–3.80)	0.20 (0.0–11.10)	< **0**.**01**	**↑**
Radiological severity visual score ≥ 8	16 (25.0%)	5 (17.86%)	3 (14.29%)	8 (53.33%)	**0**.**04**	**↑**
Mild	37 (57.81%)	16 (57.14%)	15 (71.43%)	6 (40.0%)	> 0.05	
Moderate	21 (32.81%)	11 (39.28%)	6 (28.57%)	4 (26.67%)		
Severe	4 (6.25%)	1 (3.57%)	0	3 (20.0%)		
Critical	2 (3.13%)	0 (0.0%)	0	2 (13.33%)		

Categorical variables are expressed as counts and percentage in parenthesis. Continuous variables are shown as median and range in parenthesis. Significant *p* values (< 0.05) are showed in bold type

Automatic segmentation on CT images based on Hounsfield unit was performed on 74 patients because the AI tool was not able to segment automatically GGO, consolidations or emphysema in 24/98 (24.5%) cases (Fig. 1). Therefore, radiological severity visual score on CT for COVID-19 disease was provided for these 74 patients.

Concomitant emphysema was documented in 25/74 (33.8%) patients. Consolidations, emphysema and residual healthy lung parenchyma volumes showed differences statistically significant in the three groups of patients based on outcome. Consolidations and emphysema volumes are increased in hospitalized patient in critical condition or died while residual healthy lung parenchyma is decreased (Table 2). On the other hand, GGO volumes did not affect the patients outcome; this result is explained by the high percentage of GGO in all patients groups.

### Univariable and Logistic multivariate regression analysis

Tables 3 and 4 summarize univariable and multivariable logistic regression analysis results. In the multivariable analysis obtained with only clinical parameters, significant predictors of the outcome were age > 61 years (OR 3.93; *p* < 0.01), SpO2 ≤ 93% (OR 2.73; *p* < 0.01) and ALT > 29.0 IU/L (OR, 4.28; *p* < 0.001). In the multivariable analysis obtained with only CT parameters, significant predictors of the outcome were emphysema volume > 0.10% (OR 4.43; *p* < 0.001), residual healthy lung parenchyma volume ≤ 70.20% (OR 4.40; *p* < 0.001) and consolidation volume > 1.40% (OR 5.69; *p* < 0.001). In the multivariable analysis obtained with clinical and CT parameters, significant predictors of the outcome were age > 61 years (OR 3.62; *p* < 0.001), ALT > 29.0 IU/L (OR 2.76; *p* < 0.001), emphysema volume > 0.10% (OR 2.73; *p* < 0.01), residual healthy lung parenchyma volume ≤ 70.20% (OR 3.54; *p* < 0.001) and consolidation volume > 1.40% (OR 3.19; *p* < 0.01). The highest value of *R*-squared (*R*^2^ = 0.93) was obtained by the model that combines clinical/laboratory findings at CT volumes (Table 4).

**Table 3:** Univariable analysis for the relationship between baseline clinical and CT parameters to predict patient’s outcome

Univariable analysis	Coefficients	Odds ratio	*p* value
Age > 61	2.21	11.85	≪ **0**.**001**
SpO2 (%) ≤ 93	1.49	5.19	≪ **0**.**001**
HS CRP (mg/dL) > 8.41	2.04	10.16	≪ **0**.**001**
Leukocyte (10^3^ µL) > 6.20	1.85	7.98	≪ **0**.**001**
Neutrophils (%) > 77.55	1.98	9.36	≪ **0**.**001**
LDH (IU/L) > 323	2.02	9.75	≪ **0**.**001**
d-dimer (mg/L) > 0.96	2.10	8.82	≪ **0**.**001**
Troponin (mg/mL) > 9.90	2.24	7.51	≪ **0**.**001**
Creatinine (mg/dL) > 0.90	2.04	9.75	≪ **0**.**001**
Azotemia (mg/dl) > 35.0	2.17	10.87	≪ **0**.**001**
ALT (IU/L) > 29.0	2.09	9.38	≪ **0**.**001**
AST (IU/L) > 38.0	2.05	8.99	≪ **0**.**001**
Bilirubin (mg/dl) > 0.50	1.87	7.97	≪ **0**.**001**
Residual healthy lung parenchyma (%) ≤ 70.20	1.55	5.54	≪ **0**.**001**
Consolidation (%) > 1.40	1.97	7.43	≪ **0**.**001**
Emphysema (%) > 0.10	2.16	7.72	≪ **0**.**001**
Radiological severity visual score ≥ 8	2.23	4.81	≪ **0**.**001**

*IU* international unit

**Table 4:** Logistic multivariate analysis for the relationship between baseline clinical qualitative and quantitative CT parameters to predict patient’s outcome

	Coefficients	Odds ratio	*p* value	*R*-squared (*R*^2^)
*Clinical/laboratory findings*				
Age > 61	0.81	3.93	≪ ** 0**.**01**	0.86
SpO2 (%) ≤ 93	0.44	2.73	≪ **0**.**01**	
HS CRP (mg/dL) > 8.41	0.36	1.93	> 0.05	
Leukocyte (10^3^ µL) > 6.20	0.08	0.38	> 0.05	
Neutrophils (%) > 77.55	0.25	1.24	> 0.05	
LDH (IU/L) > 323	0.14	0.69	> 0.05	
d-dimer (mg/L) > 0.96	0.26	1.42	> 0.05	
Troponin (mg/mL) > 9.90	− 0.10	− 0.45	> 0.05	
Creatinine (mg/dL) > 0.90	0.25	1.24	> 0.05	
Azotemia (mg/dl) > 35.0	0.33	1.59	> 0.05	
ALT (IU/L) > 29.0	0.77	4.28	≪ **0**.**001**	
AST (IU/L) > 38..0	− 0.11	− 0.50	> 0.05	
Bilirubin (mg/dl) > 0.50	0.20	1.22	> 0.05	
*CT findings*				
Residual healthy lung parenchyma (%) > 70.20	0.84	4.40	≪ **0**.**001**	0.78
Consolidation (%) > 1.40	1.25	5.69	≪ **0**.**001**	
Emphysema (%) > 0.10	1.06	4.43	≪ **0**.**001**	
Radiological severity visual score ≥ 8	0.48	1.44	> 0.05	
*Clinical/laboratory and CT findings*				
Age > 61	0.80	3.62	≪ **0**.**001**	0.93
SpO2 (%) > 93	0.05	0.25	> 0.05	
HS CRP (mg/dL) > 8.41	0.29	1.39	> 0.05	
ALT IU/L > 29.0	0.57	2.76	≪ **0.01**	
Emphysema (%) > 0.10	0.61	2.76	≪ **0**.**01**	
Residual healthy lung parenchyma (%) ≤ 70.20	0.73	3.54	≪ **0**.**001**	
Consolidation (%) > 1.40	0.74	3.19	≪ **0**.**01**	

*IU* international unit

**Fig. 3:**
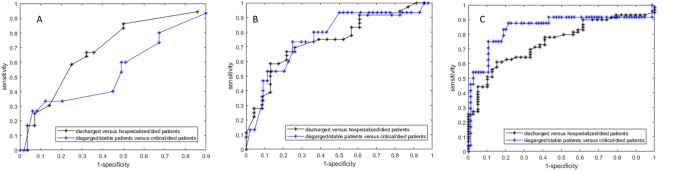
ROC curve for Clinical/Laboratory findings model (A), CT volumes model (B), clinical/laboratory ad CT findings model

Table 5 reports the performance analysis in terms of sensitivity, specificity, positive predictive value, negative predictive value and accuracy obtained by ROC analysis for clinical/laboratory findings model, CT model and clinical/laboratory and CT findings model to identify discharged versus hospitalized/died patients or to identify discharged/stable patients versus critical/died patients. Figure 3 reports the ROC curves. The highest accuracy was obtained by clinical/laboratory and CT findings model with a sensitivity, a specificity and an accuracy, respectively, of 88%, 78% and 81% to predict discharged/stable patients versus critical/died patients.

**Table 5:** ROC analysis results

	AUC	Sensitivity	Specificity	Positive predictive value	Negative predictive value	Accuracy
*Clinical/laboratory model*						
To identify discharged versus hospitalized/died patients	0.57	0.86	0.50	0.69	0.74	0.70
To identify discharged/stable patients versus critical/died patients	0.47	0.33	0.88	0.45	0.81	0.75
*CT model*						
To identify discharged versus hospitalized/died patients	0.70	0.58	0.87	0.88	0.57	0.69
To identify discharged /stable patients versus critical/died patients	0.75	0.73	0.75	0.50	0.89	0.75
*Clinical/laboratory and CT findings model*						
To identify discharged versus hospitalized/died patients	0.75	0.61	0.85	0.86	0.59	0.70
To identify discharged/stable patients versus critical/died patients	0.83	0.88	0.78	0.57	0.95	0.81

## Discussion and conclusions

Several authors have reported in the recent literature the role of chest X-rays and of CT in patients affected by COVID-19 disease, the evolution of these features over time and the radiologist’s performance to differentiate COVID-19 from other viral infections [23–29]. In these studies were reported the main characteristics of COVID-19 viral pneumonia on chest CT images: peripheral GGO and nodular or mass-like GGO with a distribution bilateral and multilobar [26]. Guan et al. [27] reported as main CT patterns GGO (56.4%) and bilateral patchy shadowing (51.8%). In our cohort, GGO and consolidations were the main CT patterns: a variable combination of GGO and consolidations was found in 87.8% of patients. Moreover, CT COVID-19 disease was prevalently bilateral (77.6%) with peripheral distribution (74.5%) and localizations in multiple lobes (52.0%) were found. Crazy paving pattern was reported in the 56.1% of patients and “reversed halo” sign in the 20.4% of patients.

As for the visual quantification of the GGO and the consolidation extension, our data document a correlation between the radiological severity visual score (≥ 8) and the outcome while no correlation was found considering individually the patients groups mild, moderate, severe and critical due to the similar percentage of radiological score in all groups.

Several methods of disease extent quantification at chest CT have been proposed, including the extent of emphysema, pulmonary fibrosis and acute respiratory distress syndrome (ARDS) [30–35]. CT score of the burden of lung disease was previously reported as a risk factor for mortality in ARDS [30]. However, there are scarce data on the prognostic value of CT in COVID-19. A visual semi-quantitative quantification of disease extent at CT correlated with clinical severity [36].

Colombi et al. [37] reported that in patients with confirmed COVID-19 pneumonia, visual or software quantification the extent of CT lung abnormality was predictors of ICU admission or death. They reported that the proportion of well-aerated lung assessed by chest CT obtained in the emergency department was associated with better prognosis for patients with COVID-19 pneumonia independent of other clinical parameters.

Considering the substantial rate of ARDS in COVID-19 patients, we assessed by means of regression models the relationships between baseline clinical data and disease lung involvement on baseline chest CT and we quantified the residual healthy lung parenchyma volume to predict prognosis in patients with COVID-19 pneumonia.

In our study, we reported that no symptoms and no comorbidities determined differences statistically significant in terms of patient outcome. SpO2 was significantly lower in patients hospitalized in critical conditions or died while age, HS CRP, Leukocyte count, neutrophils, LDH, d-dimer, troponin, creatinine and azotemia, ALT, AST and bilirubin values were significantly higher. The results of the present study showed that the proportion of residual healthy lung parenchyma volume ≤ 70.20% with a proportion of emphysema volume > 0.10% and a proportion of consolidation volume > 1.40% reached a sensitivity, a specificity and an accuracy of 73%, 75% and 89% to predict discharged/stable patients versus critical/died patients.

Instead, clinical/laboratory and CT findings model obtained a sensitivity, a specificity and an accuracy, respectively, of 88%, 78% and 81% to predict discharged/stable patients versus critical/died patients.

Our results were in accordance with the findings of Raun et al. [38] that reported a significant difference in age between the death group and the discharge group (*p* < 0.001) and significant differences in white blood cell counts, absolute values of lymphocytes, platelets, albumin, total bilirubin, blood urea nitrogen, blood creatinine, myoglobin, cardiac troponin, C-reactive protein (CRP) and interleukin-6 (IL-6) between the two groups.

The main limitation of the present study is the nature retrospective and monocentric of the study conducted on a cohort of symptomatic hospitalized patients from an area of high epidemiological risk and with a high pre-test probability of COVID-19 infection.

In conclusion, our results suggest that software-based quantification of the consolidation, emphysema and residual healthy lung parenchyma on chest CT was independent predictors of COVID-19 patient’s outcome. Moreover, also a visual radiological severity visual score ≥ 8 was predictor of patient’s outcome. Both CT visual quantitative analysis and CT computerized software-based assessment of the lung involvement by COVID-19 may be useful for routine patient management to evaluate the distribution and the severity of COVID-19 pneumonia.
